# Timed Up-and-Go Test Using Instrumented Insoles to Monitor Tibial or Malleolar Fracture Healing in Patients With Healing Fractures or Nonunion: Longitudinal and Cross-Sectional Exploratory Study

**DOI:** 10.2196/92493

**Published:** 2026-09-21

**Authors:** Elke Warmerdam, Lea-Marie Burger, Bergita Ganse

**Affiliations:** 1 Innovative Implant Development (Fracture Healing), Departments and Institutes of Surgery, Saarland University, Kirrberger Str. 100, Building 57, Homburg, Saarland, 66421, Germany, +49 68411631570

**Keywords:** mobility, nonunion, tibia, wearables, rehabilitation, mHealth, digital medicine

## Abstract

**Background:**

The timed up-and-go (TUG) test is a mobility-related functional task commonly performed in clinical practice for a variety of clinical applications. By instrumenting the TUG test with sensors, creating an instrumented TUG (iTUG), additional parameters can be extracted, which may add clinical value. Instrumented insoles are among the most promising wearable devices that are currently being explored for clinical purposes.

**Objective:**

The study aims to assess additional parameters obtained by instrumenting the TUG test with pressure-sensing insoles for monitoring fracture healing and to analyze whether these parameters reveal differences between patients with and without union of tibial or malleolar fractures.

**Methods:**

Patients with tibial or malleolar fractures and matched controls were enrolled. Patients’ TUG test data were collected as follows: (1) longitudinally—at 6 weeks, 3 months, and 6 months after surgery—and (2) cross-sectionally—at 6 months after surgery when patients were referred for nonunion treatment. All participants performed the TUG test using instrumented insoles. TUG tests were video-recorded and analyzed in terms of the time taken to complete each part of the TUG test (conventional TUG). The insole data-derived outcome measures were maximal force during sit-to-stand and stand-to-sit movements, stance time and stride time during walking, and asymmetry of the previously mentioned parameters (iTUG).

**Results:**

Twenty-seven patients with fracture, a similar number of healthy adults, and 9 patients with fracture nonunion were assessed. All TUG parameters improved throughout the healing process (all *P*<.001). The greatest improvements were detected between 6 weeks and 3 months (all *P*<.001). Among the classical TUG parameters, between 3 and 6 months, only the total TUG time, walking time, stand-to-sit time, and total number of steps significantly improved (*P*=.004, *P*=.01, *P*=.03, and *P*=.002). Based on the additional iTUG measures, 6 months after surgery, the loading of the injured side was reduced in patients compared with healthy controls during sit-to-stand and stand-to-sit movements (*P*<.001). Six months after surgery, patients with fracture nonunion had significantly poorer performance in terms of all of the TUG test parameters, except for stance time, than patients with fracture union.

**Conclusions:**

This study indicates the potential for using functional outcomes to identify patients at risk of nonunion. Additional tasks and functional outcomes require further investigation.

## Introduction

### Fracture Nonunion

Bone fracture nonunion is a serious complication associated with high socioeconomic costs [1]. It is estimated that diaphyseal nonunion of the tibia and femur occurs in 4.6% to 8% of patients following intramedullary nailing of closed fractures performed in accordance with current standards, whereas the risk is even higher in open fractures [1]. Risk factors for nonunion include severe fractures (eg, open fractures and multiple fractures), a high BMI, smoking, and alcoholism [2]. While women experience fractures more frequently, men are more prone to nonunion [2]. The nonunion rate is greatest in the navicular bone, tibia and fibula, and femur [2]. In routine clinical practice, radiographs are used to monitor fracture healing. Considering that delayed healing can lead to nonunion, patients are often monitored for an extended period, and interventions such as ultrasound stimulation or shock wave therapy are sometimes prescribed with the aim of inducing union [3]. Revision surgery with a bone graft is usually arranged once healing within a reasonable time seems unlikely [4]. Considering that mineralization of the callus in and around the fracture gap does not take place immediately, radiographs can only show bone healing with a time delay [5]. Moreover, radiographs require X-rays, which are carcinogenic [6]. To find better alternatives, functional outcome measures, such as patient-reported outcome measures, have been suggested to monitor fracture healing [7-9]. However, no correlation was shown between differences in adverse event rates and differences in patient-reported outcome measures in orthopedic trauma randomized controlled trials [10]. Although analyses of movement quality are rarely performed in daily clinical practice for the quantification of actual physical function, they may be suitable for predicting fracture nonunion, as discussed in the literature [11-13].

### The TUG Test

The timed up-and-go (TUG) test is a simple, short functional assessment that covers multiple mobility-related aspects. It has high reliability and validity [14]. This test includes rising from a chair, walking (straight and turning), and sitting down. The outcome measure of this test is the time needed to complete the test. When the TUG test is video-recorded, additional outcome measures can be assessed via video analysis, including the time it takes to perform each subtask and the number of steps [14]. The TUG test is known to be a reliable measure in different populations, such as older adults and patients with a history of a stroke, spinal cord injury, or cerebral palsy [15]. Endurance training, resistance training, and physical therapy are known to reduce the time it takes for older adults, patients with chronic stroke, and patients with Parkinson disease to perform the TUG test [16-18]. The time it takes to complete the TUG test can provide information about future fracture risk above that provided by bone mineral density, as it has been shown in 2 studies [19,20].

In recent decades, sensors, such as inertial measurement units and instrumented insoles, have been increasingly used during TUG tests [21,22]. These additions make it possible to extract additional outcome parameters, such as gait parameters [23,24], joint kinematics [25], angular velocities [26], and accelerations [27]. Compared to the traditional TUG test, the predictive value of the instrumented TUG (iTUG) was higher for fall prediction owing to the additional extracted, mainly temporal and velocity, parameters [22]. Patients with idiopathic normal-pressure hydrocephalus, Parkinson disease, and orthopedic conditions may complete the iTUG test as a means to screen for treatment and disease-related changes in mobility [21]. The results of the iTUG test after total knee or hip joint replacement improved over the course of rehabilitation and provided additional information [28,29]. However, it is not yet clear whether changes during fracture healing can similarly be quantified with the iTUG test or whether there are advantages of the iTUG test over the conventional TUG test.

### Improvements After Injury

Instrumented gait analysis with motion capture systems or wearables can be used to quantify improvements in the gait pattern throughout the healing process of tibial and malleolar fractures [11,30]. There is also potential to analyze other mobility-related movements, such as standing and rising from a chair. In particular, the analysis of the vertical ground reaction force of these movements is of interest, as patients can choose how to distribute the load between the injured and uninjured sides, which is not the case with walking once they can walk without crutches. When patients begin to walk without crutches after the injury, the improvements in maximal loading during walking are rather small because they will always reach loading values of 100% of their body weight during the single-support phase. The normative values for loading lie between 100% of body weight at slow speeds and 125% at fast speeds [31]. Therefore, other movements might be even more sensitive for monitoring fracture healing at later stages of healing.

### Hypotheses

Hence, this study analyzed whether the TUG test performed with instrumented insoles can be used to monitor the healing status of tibial and malleolar fractures and whether it has advantages over the conventional TUG test. This information could aid in the process of deciding when to revise or intervene during the fracture healing process. It was hypothesized that (1) longitudinal changes in mobility throughout the tibial fracture healing can be assessed in terms of iTUG test results; (2) 6 months after a tibial fracture, the performance of several iTUG parameters returns to that of sex- and age-matched healthy controls; and (3) several parameters measured in the iTUG test can distinguish between patients with and without union 6 months after a tibial fracture.

## Methods

This longitudinal, observational, exploratory study was registered with the German Clinical Trials Register (DRKS-ID: DRKS00025108). The STROBE (Strengthening the Reporting of Observational Studies in Epidemiology) checklist is provided as a download (Checklist 1).

### Participants

Patients with tibial or malleolar fractures who underwent surgical treatment at Saarland University Hospital between February 2022 and January 2024 were included and assigned to the longitudinal patient group. Patients who were younger than 18 years could not provide informed consent, had limited mobility before the fracture, had other injuries that could affect mobility, or were pregnant were excluded. Patients were recruited during their hospital stay after undergoing surgical treatment for their fracture. Radiographs in 2 planes obtained 6 months after the fracture were examined by experienced trauma surgeons to determine whether the fracture had healed.

In addition, a group of patients with fracture nonunion was recruited from those treated in the outpatient nonunion clinic. As only less than 10% of patients develop nonunion, a longitudinal study would take several years to reach a sufficient number of patients with fracture nonunion. At Saarland University Hospital, where this study was conducted, patients who were originally treated in other hospitals can present to seek advice and further treatment for their nonunion in the specialized nonunion outpatient clinic, which serves a larger region. To increase the number of patients with fracture nonunion in this study, the authors added a cross-sectional group of patients from this outpatient group, for whom longitudinal measurements were impossible, because they had initially been treated elsewhere. These patients received only one measurement, as they underwent their revision surgery shortly after, and their further treatment was again conducted at the hospital where they had originally been treated. The inclusion criteria for this cross-sectional nonunion group were surgery 4 to 10 months earlier and nonunion as diagnosed by an experienced trauma surgeon. The exclusion criteria were below 18 years of age, inability to provide informed consent, limited mobility before the fracture, other injuries that could affect mobility, or pregnancy.

Healthy controls were sex- and age-matched to the longitudinal patient group. These participants were recruited among the spouses of the patients, hospital employees, and acquaintances of the study team and were measured between February 2022 and April 2024. Healthy participants were excluded if they were below 18 years of age, were unable to provide written informed consent, or had limited mobility. They underwent only a single measurement, as changes were not expected over time without a fracture.

### Study Protocol

Patients in the longitudinal patient group were assessed during each outpatient visit, which was usually scheduled approximately 6 weeks, 3 months, and 6 months after surgery. The first visit had to take place between 30 and 60 days after surgery, the second visit between 75 and 105 days, and the third visit between 135 and 235 days. Only patients who took part in all 3 assessments within these time frames were included in the analysis. In the patients with fracture nonunion group and the healthy control group, only one measurement was performed. During the experiment, all participants wore instrumented insoles (OpenGO insoles, Moticon GmbH) containing 16 pressure sensors in both shoes. Before the start of each measurement, the insoles were fitted in accordance with the shoe size of each participant and calibrated to each participant’s body weight using the insole software calibration function. All patients and participants wore sports shoes with a soft sole. The participants were asked to sit down on a wooden chair measuring 45.7 centimeter in height with their back against the backrest of the chair. At the start of the TUG test, they had to rise from the chair, walk for 3 meters, turn around a marked spot on the floor, walk back to the chair, and sit down again with their back against the backrest. They were instructed to perform this assessment at their preferred speed and to turn in their preferred direction. If patients still used crutches in their daily lives, they were allowed to use crutches to perform the task. The tests were video-recorded (HERO7 Silver, GoPro Inc).

### Data Analysis

The total TUG time, time per subtask, and the number of steps were extracted from the video recordings using the open-source software Media Player Classic–Home Cinema (MPC-HC, version 1.7.13; open source, MPC Community Forum) based on Savoie et al [32], but with slight adjustments. The first video frame in which the patient moved the upper part of the trunk forward marked the start of the TUG test. The frame in which they started to walk (heel rise, which was followed by toe-off) marked the start of the next subtask. The moment the patient placed the supporting leg for the first single step, in which the hips or legs began a turning movement in their preferred turn direction, marked the start of the turn. The end of the turn was analogously defined as the touchdown of the swing leg after the last individual step in which the hips or legs were still in a turning motion. Since the turn and sit-down movements at the end of the TUG test are generally performed in combination, the start of this stand-to-sit movement was defined as the landing of the supporting leg on the first single step, when the hips or legs were again in a turning movement. The test was completed when the patient’s back touched the backrest. The numbers of left and right steps were counted from the start of the TUG test (forward movement of the trunk) until the end of the TUG test (patient’s back touched the backrest) and then summed.

**Figure 1.**
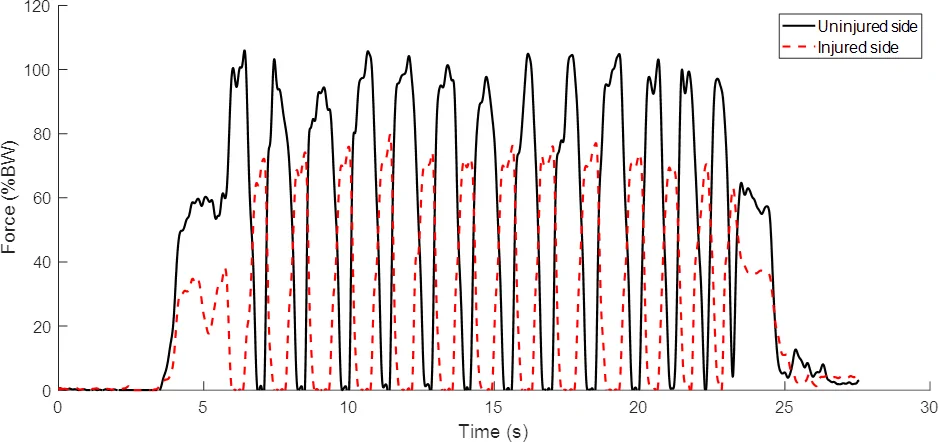
Vertical ground reaction forces of a 76-year-old female patient with a malleolar fracture, measured 76 days after surgery during the timed up-and-go test. BW: body weight.

The raw data from the insoles were downloaded with the manufacturer’s software. A custom MATLAB (version 2021b; MathWorks) script was written to extract the sit-to-stand and stand-to-sit movements and the steps. Data were filtered with a fourth-order Butterworth filter with a 6-Hertz cutoff frequency. The sit-to-stand and stand-to-sit movements were manually detected in the ground reaction force curves of both feet, whereafter the peak in the signal was detected and used as the maximal force during sit-to-stand and stand-to-sit movements (see Figure 1 for an example of the forces during the TUG test). The manual detection of these movements was performed to ensure that these movements were also detected when no force was applied to the insole on the injured side or when the data from both feet were not correctly synchronized by the software. A threshold of 30 N in the vertical force data was used to detect the initial and final contact of each step that occurred between the sit-to-stand and stand-to-sit movements. The vertical force data were normalized to the percentage of body weight to allow for comparisons between participants. From the sit-to-stand and stand-to-sit movements, the maximal force was extracted for both the injured and uninjured legs. From the walking phase, the maximal force and stance and stride times were extracted for each stride. An average of these parameters was calculated per side. Additionally, the asymmetry of all of these parameters was calculated as a percentage using the following equation:


$$Asymmetry=\frac{\left(uninjured\;side-injured\;side\right)}{0.5\times \left(uninjured\;side+injured\;side\right)}\times 100$$

### Statistical Analyses

Statistical analyses were performed with JASP (version 0.17.3.0). The data were tested for a normal distribution with the Shapiro-Wilk test. Differences in demographics between patients with union and healthy controls, as well as between patients with union and those without union, were assessed with an independent *t* test in the case of a normal distribution; otherwise, a Mann-Whitney *U* test was used. A repeated-measures ANOVA was performed to analyze whether the extracted parameters of the longitudinal patient group improved throughout the healing phase, with the extracted TUG parameters as the within-subject factors. Effect sizes were calculated according to eta-squared. Post hoc testing was performed with Bonferroni corrections. The parameters assessed at 6 months after surgery in the group of longitudinal patients with union were compared with those of healthy controls via an independent *t* test or a Mann-Whitney *U* test in cases of violation of normality. Additionally, the longitudinal patient group at 6 months was compared with patients with nonunion at 6 months after surgery via an independent *t* test or a Mann-Whitney *U* test in the absence of a normal distribution. Effect sizes were calculated using the Cohen *d* method. The magnitude of Cohen *d* was categorized as follows: small (*d*=0.2), medium (*d*=0.5), and large (*d*≥0.8). Significance was set at a *P* value <.05 for all tests.

### Ethical Considerations

The study protocol was approved by the Institutional Review Board of the Saarland Medical Board (Ärztekammer des Saarlandes, Germany, application number 30/21). Written informed consent was obtained from all participants before the start of the measurements. Participants had the opportunity to opt out at any time without needing to provide reasons. This observational, prospective longitudinal cohort study was conducted in accordance with the Declaration of Helsinki. The data were pseudonymized. The participants did not receive any financial or nonfinancial compensation for their participation in the study.

## Results

### Demographics

Twenty-seven patients (Table 1) with union of a tibial or malleolar fracture were assessed 3 times during the healing process. Twenty-seven healthy adults, matched for sex and age with the longitudinal patient group, served as the control group. Additionally, 9 patients with nonunion were included in the nonunion group. There were no significant differences in demographics between the longitudinal patient group and the control group or between the longitudinal patient group and the patients with fracture nonunion group (Table 1).

**Table 1.** Demographics of all groups.

Parameter	Longitudinal patients with union	Healthy controls	Patients with fracture nonunion	*P* value	
				Longitudinal patients vs controls	Longitudinal patients vs patients with fracture nonunion
N (% female)	27 (44)	27 (44)	9 (33)	—^a^	—
Age (y), mean (SD)	54 (13)	54 (13)	59 (18)	.92	.11
Height (cm), mean (SD)	175 (9)	173 (9)	178 (9)	.67	.52
Weight (kg), mean (SD)	83 (13)	79 (15)	80 (14)	.13	.62
BMI	27 (4)	26 (4)	25 (4)	.62	.29
Fracture site (proximal tibia/tibial shaft/distal tibia/malleolar)	9/5/1/12	—	1/8/0/0	—	—

a Not applicable.

### Longitudinal Changes in the Performance of the (Instrumented) TUG Test

**Figure 2.**
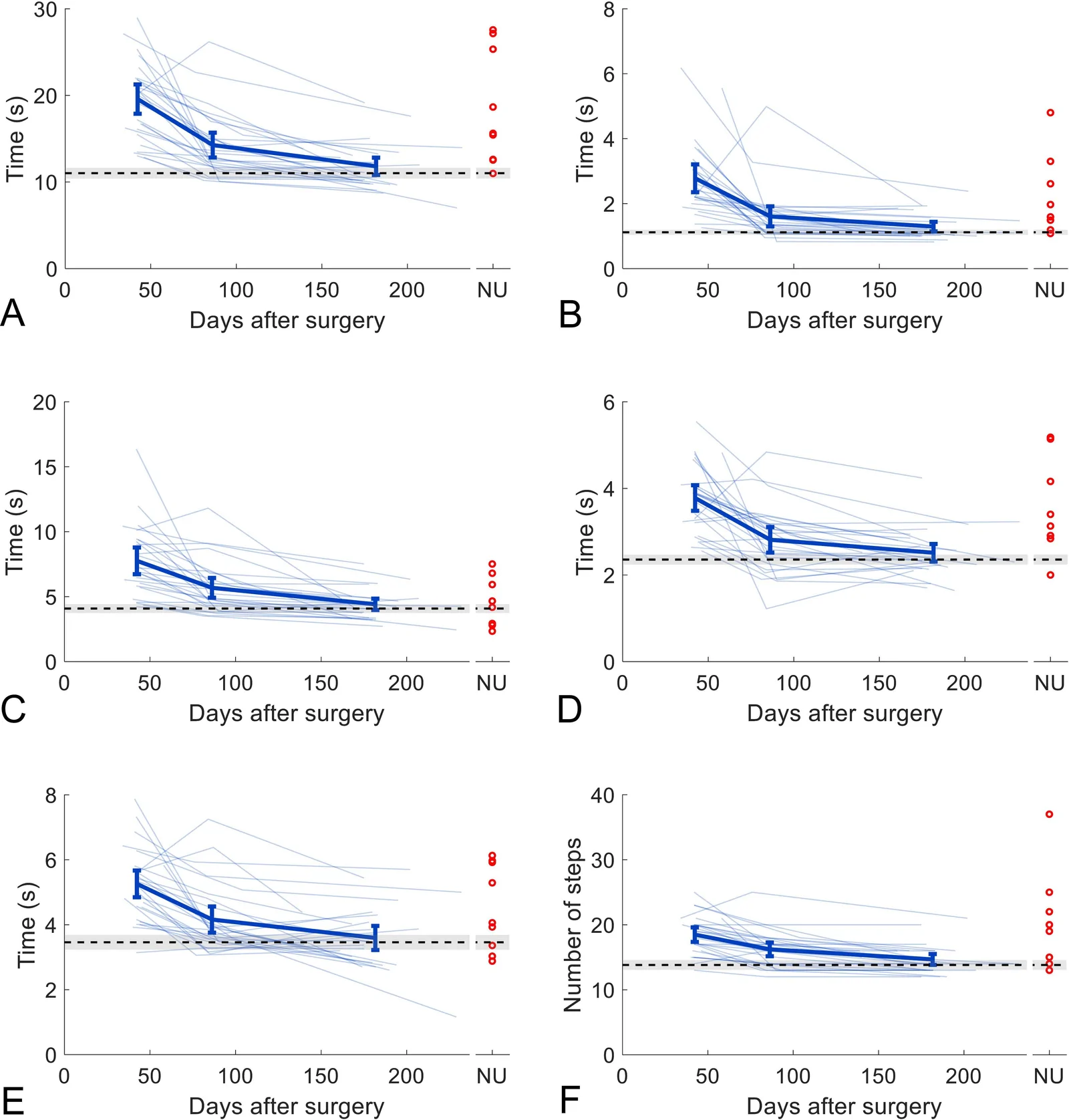
Timed up-and-go test parameters extracted from the video recordings. (A) Total time; (B) sit-to-stand time; (C) walking time; (D) turning time; (E) stand-to-sit time; (F) total number of steps. The thin blue lines represent the results for each patient, and the thicker blue line represents the average of the longitudinal patients. The whiskers show the 95% CIs of the longitudinal data. The horizontal black dotted line represents the average of the matched controls, and the 95% CIs are in light gray. The red circles are the data points of the patients with fracture nonunion. NU: nonunion group.

**Figure 3.**
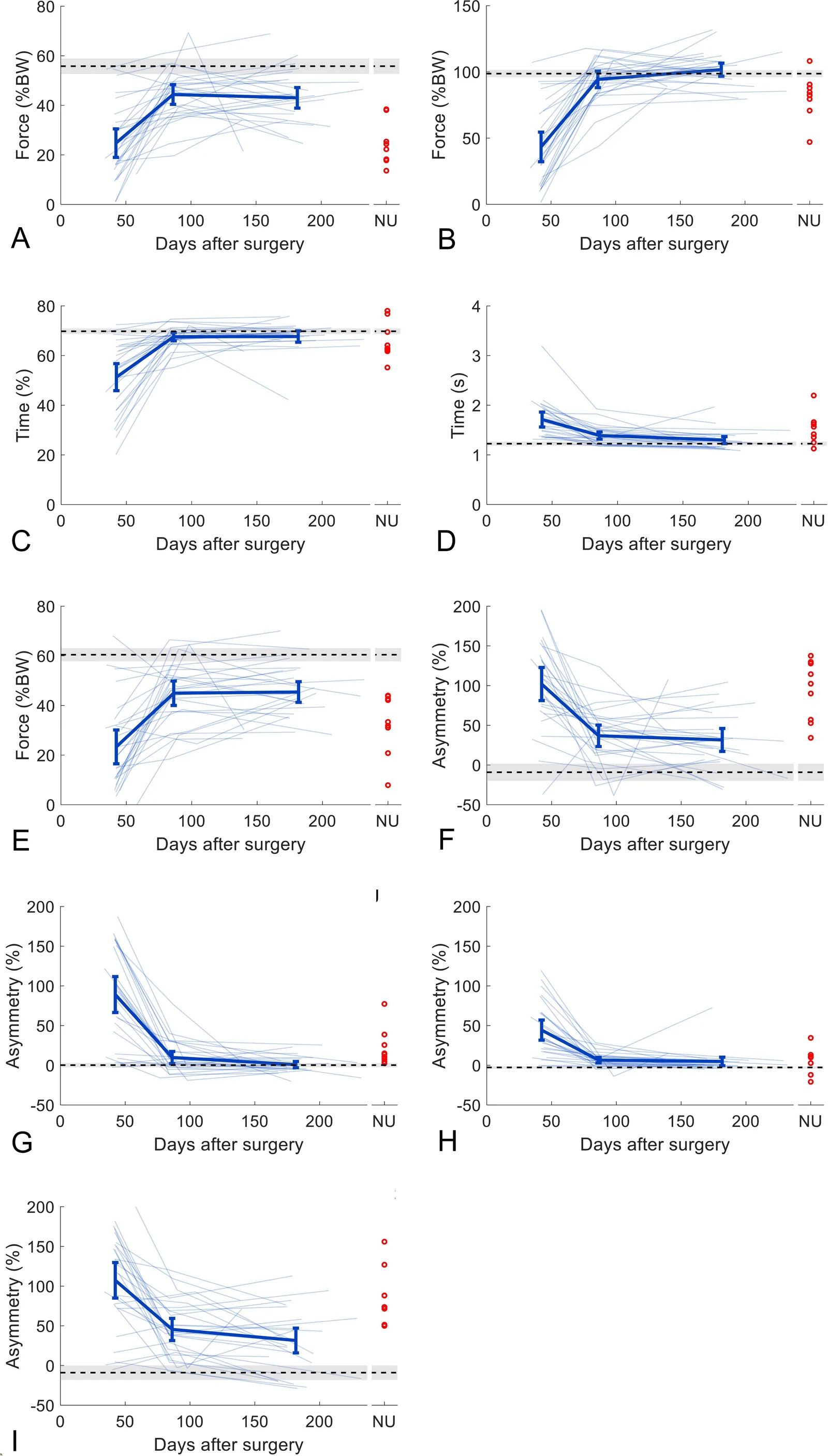
Timed up-and-go test parameters extracted from instrumented insoles. (A) Maximal force sit-to-stand; (B) maximal force walking; (C) stance time walking; (D) stride time walking; (E) maximal force stand-to-sit; (F) asymmetry maximal force sit-to-stand; (G) asymmetry maximal force walking; (H) asymmetry stance time; (I) asymmetry maximal force stand-to-sit. The thin blue lines represent the results of each patient, and the thicker blue line represents the average of the longitudinal patients. The whiskers show the 95% CIs of the longitudinal data. The horizontal black dotted line represents the average of the matched controls, and the 95% CIs are in light gray. The red circles are the data points of the patients with fracture nonunion. BW: body weight; NU: nonunion group.

The longitudinal patient group was assessed on days 42, 86, and 182, on average, after surgery. At 6 weeks after surgery, 22% (6/27) of the patients performed the test without crutches. At 3 months, this number increased to 85% (23/27), and at 6 months, none of the patients used crutches anymore. Repeated-measures ANOVA revealed significant changes in all parameters throughout healing. Post hoc tests revealed that all parameters significantly improved between 6 weeks and 3 months after surgery. Between 3 and 6 months, only the total TUG time, walking time, stand-to-sit time, and total number of steps significantly improved (Table 2 and Figures 2 and 3).

**Table 2.** Differences between time points in the longitudinal measurements (N=27).^a^

Parameter	Mean (SD)			*P* value	Effect size (η^2^)	Post hoc *P* value		
	T1^b^	T2^c^	T3^d^			T1 vs T2	T1 vs T3	T2 vs T3
TUG^e^ parameters extracted from video recordings								
Total time (s)	19.6 (4.5)	14.3 (3.8)	11.8 (2.6)	<.001^f^	0.696^f^	<.001^f^	<.001^f^	.004^f^
Sit-to-stand time (s)	2.8 (1.1)	1.6 (0.8)	1.3 (0.4)	<.001^f^	0.521^f^	<.001^f^	<.001^f^	.42
Walking time (s)	7.8 (2.7)	5.7 (2.0)	4.4 (1.2)	<.001^f^	0.549^f^	<.001^f^	<.001^f^	.01^f^
Turning time (s)	3.8 (0.8)	2.8 (0.8)	2.5 (0.5)	<.001^f^	0.553^f^	<.001^f^	<.001^f^	.22
Stand-to-sit time (s)	5.3 (1.1)	4.2 (1.1)	3.6 (1.0)	<.001^f^	0.555^f^	<.001^f^	<.001^f^	.03^f^
Total number of steps	18.5 (3.0)	16.2(2.8)	14.7 (2.2)	<.001^f^	0.602^f^	<.001^f^	<.001^f^	.002^f^
TUG parameters extracted from instrumented insoles								
Maximal force sit-to-stand (%BW^g^)	24.7 (15.2)	44.4 (10.6)	43.0 (11.0)	<.001^f^	0.493^f^	<.001^f^	<.001^f^	>.99
Maximal force walking (%BW)	43.4 (29.6)	94.3 (16.5)	101.7 (13.1)	<.001^f^	0.737^f^	<.001^f^	<.001^f^	.50
Stance time walking (%)	51.3 (14.5)	67.6 (4.4)	67.7 (6.1)	<.001^f^	0.547^f^	<.001^f^	<.001^f^	>.99
Stride time walking (s)	1.7 (0.4)	1.4 (0.2)	1.3 (0.2)	<.001^f^	0.522^f^	<.001^f^	<.001^f^	.35
Maximal force stand-to-sit (%BW)	23.3 (18.1)	44.9 (13.0)	45.4 (11.0)	<.001^f^	0.524^f^	<.001^f^	<.001^f^	>.99
Asymmetry maximal force sit-to-stand (%)	102.0 (55.3)	36.8 (35.7)	31.6 (38.4)	<.001^f^	0.471^f^	<.001^f^	<.001^f^	>.99
Asymmetry maximal force walking (%)	89.1 (60.1)	9.8 (20.3)	0.9 (10.4)	<.001^f^	0.669^f^	<.001^f^	<.001^f^	>.99
Asymmetry stance time (%)	44.3 (33.7)	6.5 (9.1)	4.9 (14.2)	<.001^f^	0.529^f^	<.001^f^	<.001^f^	>.99
Asymmetry maximal force stand-to-sit (%)	107.3 (59.3)	45.4 (37.0)	31.5 (41.3)	<.001^f^	0.540^f^	<.001^f^	<.001^f^	.55

a The results of the repeated-measures ANOVA for the longitudinal patient group with union are shown.

b T1: 6 weeks.

c T2: 3 months.

d T3: 6 months.

e TUG: timed up-and-go.

f These values represent significant results.

g BW: body weight.

The maximal force during walking had the greatest effect size, followed by the total TUG time (Table 2). The remaining effect sizes of the parameters extracted from the video recordings are comparable to those of the parameters extracted from the instrumented insoles. However, between 3 and 6 months after surgery, significant changes were observed only in the following video-based parameters: the total time, walking time, stand-to-sit time, and the total number of steps.

### Group Differences in the Performance of the TUG Test

Six months after surgery, the maximal force during the sit-to-stand and stand-to-sit movements of patients with union was significantly lower than that of the matched controls, and the asymmetry of these parameters and the stance time were significantly greater in the patients with union (Table 3). The other TUG test parameters were similar to those of healthy controls. The comparison of patients with and without union at an average of 182 and 200 days after surgery, respectively, revealed significant differences in almost all the parameters, except for stance time and stance time asymmetry (Table 3). It should be noted that 2 of the patients with fracture nonunion performed the test with crutches, whereas the patients with fracture nonunion all performed the test without crutches at 6 months after surgery. The parameters extracted from the videos showed the 3 greatest effect sizes of all parameters. These were the total time, the time walking, and the time turning. A large effect was also observed for the stand-to-sit time (Cohen *d*>0.8). Nonetheless, the effect sizes of the force-related parameters were also large, except for the asymmetry of the maximal force during walking. Among the forces, the largest effects were found for the maximal force sit-to-stand, maximal force walking, and the asymmetry in the maximal force sit-to-stand, but their effect sizes were smaller than those of the video-related parameters.

**Table 3.** Comparisons between longitudinal patients with union 6 months after surgery and sex- and age-matched healthy controls, as well as between longitudinal patients with union and patients with fracture nonunion 6 and 10 months after surgery, respectively.

Parameter	Mean (SD)			Patients with union vs controls		Patients with union vs patients with fracture nonunion	
	Union group at 6 months (N=27)	Healthy controls (N=27)	Nonunion group at 6 months (n=9)	*P* value	Effect size (Cohen *d*)	*P* value	Effect size (Cohen *d*)
TUG^a^ parameters extracted from video recordings							
Total time (s)	11.8 (2.6)	11.0 (1.6)	18.4 (6.6)	.19	−0.359	<.001^b^	−1.675^b^
Sit-to-stand time (s)	1.3 (0.4)	1.1 (0.2)	2.2 (1.2)	.06^c^	−0.299	.005^b^^,^^c^	−0.634^b^
Walking time (s)	4.4 (1.2)	4.1 (0.9)	8.0 (3.6)	.26	−0.313	<.001^b^	−1.764^b^
Turning time (s)	2.5 (0.5)	2.4 (0.3)	3.8 (1.2)	.19	−0.359	<.001^b^	−1.689^b^
Stand-to-sit time (s)	3.6 (1.0)	3.5 (0.6)	4.5 (1.3)	.55	−0.164	.03^b^	−0.851^b^
Total number of steps	14.7 (2.2)	13.8 (2.0)	19.9 (7.6)	.11^c^	−0.248	.03^b^^,^^c^	−0.477^b^
TUG parameters extracted from instrumented insoles							
Maximal force sit-to-stand (%BW^d^)	43.0 (11.0)	55.8 (8.3)	26.3 (9.7)	<.001^b^	1.313^b^	<.001^b^	1.567^b^
Maximal force walking (%BW)	101.7 (13.1)	98.7 (7.0)	81.5 (16.4)	.30	−0.286	<.001^b^	1.451^b^
Stance time walking (%)	67.7 (6.1)	69.8 (2.9)	65.9 (7.5)	.12^c^	0.246	.18^c^	0.309
Stride time walking (s)	1.3 (0.2)	1.2 (0.1)	1.5 (0.3)	.20^c^	−0.203	.02^b^^,^^c^	−0.514^b^
Maximal force stand-to-sit (%BW)	45.4 (11.0)	60.5 (7.0)	33.1 (12.2)	<.001^b^	1.623^b^	.008^b^	1.092^b^
Asymmetry maximal force sit-to-stand (%)	31.6 (38.4)	−9.1 (28.6)	94.0 (37.9)	<.001^b^	−1.202^b^	<.001^b^	−1.632^b^
Asymmetry maximal force walking (%)	0.9 (10.4)	0.2 (7.1)	21.8 (23.7)	.85^c^	−0.032	.001^b^^,^^c^	−0.704^b^
Asymmetry stance time (%)	4.9 (14.2)	−2.7 (3.7)	6.9 (15.9)	<.001^b^^,^^c^	−0.638^b^	.13^c^	−0.350
Asymmetry maximal force stand-to-sit (%)	31.5 (41.3)	−9.0 (24.2)	84.1 (36.4)	<.001^b^	−1.198^b^	.002^b^	−1.306^b^

a TUG: timed up-and-go.

b These values represent significant results of the independent *t* test.

c Mann-Whitney *U* test in case of the absence of a normal distribution.

d BW: body weight.

## Discussion

### Principal Results

All TUG test parameters, extracted from both video recordings and instrumented insoles, improved throughout the healing process of tibial and malleolar fractures. Six months after surgery, there were still significant differences between patients and healthy controls. Compared with that of matched controls, the maximal loading on the injured side was significantly lower during sit-to-stand and stand-to-sit movements. No differences in video-based parameters were detected between patients and healthy controls. Patients with fracture nonunion performed significantly worse in 13 of the 15 assessed parameters of the TUG test than patients with union at 6 months after surgery.

### Longitudinal Findings

The longitudinal patient group showed significant improvements in all the assessed parameters between 6 weeks and 3 months. However, between 3 months and 6 months, only 4 TUG parameters improved, all of which were video-based parameters. Most of the changes in the TUG test parameters occurred during the first 3 months. Similar findings were reported in patients with hip fractures, whose time to perform the TUG test mainly improved during the first 3 months [33]. For monitoring improvement over time, the conventional TUG test might be sufficient. However, based solely on the TUG test parameters extracted from the video recordings in this study, the performance of patients returned to normal within 6 months. Nonetheless, by instrumenting the TUG test, we were able to show that the maximal loading during sit-to-stand and stand-to-sit movements did not return to control-like values within 6 months. The time required to perform the TUG test returned to normal values within 6 months; however, the patients still tended to unload the injured side during the sit-to-stand and stand-to-sit movements, activities where they can choose how to distribute their load. The iTUG provided limited additional information compared to the video-based TUG, but the differences in loading during sit-to-stand and stand-to-sit movements may provide valuable information. Physical therapy should focus on reducing the asymmetry during these movements to prevent future complaints because of an asymmetric movement pattern. The asymmetric loading pattern could be used for further therapy planning.

The maximal loading parameters during sit-to-stand and stand-to-sit movements showed only very limited nonsignificant improvements between 3 and 6 months, and at 6 months, they were still significantly different from controls. It has previously been reported that the gait pattern 6 months after a tibial shaft fracture was still significantly different from that of healthy controls [34]. Nonetheless, no differences in temporal gait parameters were found in another cohort assessed 5 years after a tibial shaft fracture [35]. Therefore, the recovery time may be prolonged and last longer than 6 months.

Sit-to-stand and stand-to-sit movements might be valuable for monitoring the progress of fracture healing throughout the rehabilitation process because patients can choose how to distribute their weight between the injured and uninjured sides. Shortly after surgery, most patients receive partial-weight-bearing (20 kg) instructions for the first 6 weeks. At 6 weeks, patients are allowed to increase the loading of the leg. As soon as patients start to walk without walking aids (usually somewhere between 6 weeks and 3 months), they load at least 100% of their body weight on the injured side because of single-limb support phases during walking. Therefore, from that moment on, the improvements in maximal loading will be only minor. Since sit-to-stand and stand-to-sit movements are normally performed with loading on both legs, patients can choose to load the injured leg less during these movements. This is indeed the case in this study, as there is still considerable asymmetry between the loading of the injured and uninjured legs at 6 months after surgery during the sit-to-stand and stand-to-sit movements. It is not clear whether this is because of existing complaints such as pain or because patients are still very careful or even afraid to load the injured leg during these movements. Another possible cause is unawareness of unloading due to habituation. Both young and older healthy adults have good perception of weight distribution during the sit-to-stand task [36]. However, how patients with lower extremity injuries perceive their weight distribution is unknown. Furthermore, a cause for the lower maximal force could be related to decreases in strength and power after fracture. After a hip fracture, at least up to 13 weeks after surgery, women have reduced leg extension power on the injured side [37]. It is also known that the ground reaction force during the sit-to-stand movement is related to leg strength and power in older adults [38]. Therefore, potentially lower strength in the injured leg could also have led to the lower maximal force during sit-to-stand and stand-to-sit movements. The actual reason for less maximal loading of the injured side during these movements is crucial to planning physical therapy throughout the rehabilitation process.

### Nonunion

The differences between patients with and without union suggest that the iTUG test might have potential to identify patients at risk of nonunion earlier than is currently possible [39,40]. However, further investigation is warranted in longitudinal studies examining the relationship between retrospective TUG results and the diagnosis of union vs nonunion. There were no changes in the instrumented insole data between 3 and 6 months, and there were only small changes in the video-extracted parameters. It might be possible to identify patients at high risk of developing nonunion at 3 months, which may provide additional information when deciding whether there is an indication for revision surgery. Based on the effect sizes, the most suitable parameters would be the total time to perform the TUG test, the total walking time, the turning time, and the force-related parameters from the instrumented insoles during the sit-to-stand and stand-to-sit movements. If patients at risk of developing nonunion can be identified earlier, additional treatment could be started earlier during the rehabilitation process, potentially preventing nonunion and the accompanying decrease in quality of life [41] and increase in societal cost [42]. Other possible noninvasive treatments that are known to improve fracture healing in more than 80% of patients are extracorporeal shockwave therapy [43], pulsed electromagnetic fields [44], and low-intensity pulsed ultrasound [45].

### Alternative Tests Using Smart Insoles

Since this study has shown that sit-to-stand and stand-to-sit movements are valuable for monitoring rehabilitation progress after tibial and malleolar fractures, different assessments, such as the 5 times sit-to-stand test and the 30-second sit-to-stand test, may also be applicable for monitoring rehabilitation progress and determining whether the patients’ performance returns to that of healthy adults. These tests involve multiple consecutive sit-to-stand and stand-to-sit movements, and the changes observed between consecutive sit-to-stand movements may differ throughout the rehabilitation process. This group of parameters could have additional clinical value and should also be explored. Differences between similar repeated movements could result from fatigue or pain and are therefore expected to be greater at the beginning of the rehabilitation phase or in patients with nonunion.

The generalizability of the results cannot be judged from the present findings, but the authors believe that similar findings may be obtained from other patient collectives.

### Limitations

A limitation of this study is that different types of fractures were assessed together in the analysis. Owing to the small sample of the tibial fractures, it was not possible to analyze each fracture type separately. The different fracture types might have altered the results. However, these differences are expected to be minor. In addition, only patients without mobility limitations before the fracture were included in this study, but previous minor changes in gait and movement may have affected the findings in individual patients. It is therefore important to consider the patient’s mobility level before the fracture and surgery when monitoring rehabilitation. Overall, despite this being among the largest sample sizes for a nonunion group, the sample size is still small, and future studies should aim to collect larger sample sizes. Besides that, the patients with fracture nonunion were measured in a larger time frame (4-10 mo after surgery) and compared to patients with union whose measurements were taken about 6 months after surgery. This difference might have led to a chronological bias. However, since all patients with fracture nonunion had a persisting fracture gap, both groups had a similar fracture-healing status, and the effect is therefore expected to be small. Due to the small sample size, there is a risk of bias due to multiple analyses. It should be noted that muscle strength measurements are impossible in patients with fracture, as they pose a risk to the healing fracture and may result either in refracture or in implant failure. Instrumented insoles have been criticized for delivering estimates rather than measurements due to measurement error. Data have been shown to be influenced by age, body weight, body height, hand grip strength [46], the walking slope [47], and the walking surface [48]. The data delivered by instrumented insoles may be less accurate than the data recorded by force plates, and this fact should be acknowledged when interpreting the results of this study. In future studies, the pain level could be assessed each time, which was not done in this study, as the authors frequently observe patients with a nonunion who do not report any pain. However, to quantify these experiences, future studies could include such questions.

### Conclusions

Both the conventional TUG test and the iTUG test can be used to monitor functional changes throughout the healing process of tibial and malleolar fractures. Instrumenting the TUG test with pressure-sensing insoles provided additional information, which made it possible to detect differences in loading during sit-to-stand and stand-to-sit movements between patients assessed 6 months after their surgery and controls. Most of the extracted TUG test parameters differed between the patients with and without union 6 months after surgery. These differences could be detected with both the conventional TUG test and the iTUG test. The TUG test might have the potential to identify patients at risk of developing nonunion earlier. This study indicates the potential of using functional outcomes to identify patients at risk of nonunion. Further investigation of additional tasks and functional outcomes is needed.

## Supplementary material

10.2196/92493 Checklist 1 STROBE checklist.

## Abbreviations

iTUG: instrumented timed up-and-go
MPC-HC: Media Player Classic–Home Cinema
STROBE: Strengthening the Reporting of Observational Studies in Epidemiology
TUG: timed up-and-go
